# Minimally Invasive Techniques to Accelerate the Orthodontic Tooth Movement: A Systematic Review of Animal Studies

**DOI:** 10.1155/2015/608530

**Published:** 2015-12-31

**Authors:** Irfan Qamruddin, Mohammad Khursheed Alam, Mohd Fadhli Khamis, Adam Husein

**Affiliations:** ^1^Orthodontic Department, Baqai Medical University, P.O. Box 2407, Karachi, Pakistan; ^2^Orthodontic Unit, School of Dental Sciences, Universiti Sains Malaysia, Health Campus, Kota Bharu, Kelantan, Malaysia; ^3^Forensic Dentistry Unit, School of Dental Science, Universiti Sains Malaysia, Health Campus, Kota Bharu, Kelantan, Malaysia; ^4^Prosthodontic Unit, School of Dental Sciences, Universiti Sains Malaysia, Health Campus, Kota Bharu, Kelantan, Malaysia

## Abstract

*Objective*. To evaluate various noninvasive and minimally invasive procedures for the enhancement of orthodontic tooth movement in animals.* Materials and Methods*. Literature was searched using NCBI (PubMed, PubMed Central, and PubMed Health), MedPilot (Medline, Catalogue ZB MED, Catalogue Medicine Health, and Excerpta Medica Database (EMBASE)), and Google Scholar from January 2009 till 31 December 2014. We included original articles related to noninvasive and minimally invasive procedures to enhance orthodontic tooth movement in animals. Extraction of data and quality assessments were carried out by two observers independently.* Results*. The total number of hits was 9195 out of which just 11 fulfilled the inclusion criteria. Nine articles were good and 5 articles were moderate in quality. Low level laser therapy (LLLT) was among the most common noninvasive techniques whereas flapless corticision using various instruments was among the commonest minimally invasive procedures to enhance velocity of tooth movement.* Conclusions*. LLLT, low intensity pulsed ultrasound (LIPUS), mechanical vibration, and flapless corticision are emerging noninvasive and minimally invasive techniques which need further researches to establish protocols to use them clinically with conviction.

## 1. Introduction

The major concern of most of the patients going for orthodontic treatment is to improve their dentofacial esthetics while oral health benefits are secondary concerns [1]. However like other interventions orthodontic treatment with fixed appliances also poses some inherent complications and risks. These undesirable outcomes of the treatment are either due to excessive force exerted on the tooth in order to achieve movement or with difficulty in brushing and plaque accumulation around brackets [2, 3]. Irrespective of the reason, adverse effects of treatment are directly proportionate to the duration of treatment. Currently the duration of orthodontic treatment with fixed braces is 2 to 3 years on average [4, 5]; however the patient does not want more than 1.5 years [6]. Prolonged treatment duration is also detrimental to the productivity of a national healthcare system and private practices [7]; therefore accelerating the tooth movement and shortening the treatment duration have always been an issue of concern for patients as well as for orthodontists [8].

There are two basic ways to reduce the treatment duration (Table 1). One approach is by making the treatment mechanics more efficient, for example, use of low friction and self-ligating brackets [9, 10], preformed robotic archwires [11, 12], and use of microimplants [13, 14].

Another approach involves interventions to increase the velocity of orthodontic tooth movement by enhancing the bone remodeling. This intervention can be classified into three categories: (1) use of certain biochemical, (2) mechanical or physical stimulation of the alveolar bone which includes the use of cyclic vibration [15], magnets [16], or direct electrical current [16], and (3) surgical interventions to accelerate tooth movement [17].

Local administration of biochemical such as dihydroxyvitamin D3 (1,25-(OH)2D3) [18], parathyroid hormone [19], prostaglandin E2 (PGE2) [20], or osteocalcin [21] has systematic effects on body metabolism; therefore they are difficult to use for orthodontic tooth movement. Electric and pulsed electromagnetic field has no convincing evidence to be an effective modality for rapid movement [22].

Surgical procedures that enhance tooth movement involve alveolar corticotomies, rapid canine retraction, or dental distraction. These are highly invasive procedures associated with postoperative morbidity and harmful effects on periodontal tissues; thus the patient's acceptance of the procedure is low [23].

Hence the researchers are always looking for minimally invasive methods that enhance the orthodontic tooth movement and are also well accepted by the patients because of minimal side effects and low cost. Low level laser therapy [24] has shown some evidence of being effective in acceleration of tooth movement in humans and also been reviewed systematically [25]. However the need is to bring the researcher's attention towards all other techniques used in animal based researches on the subject so that there is further progress in the development of minimally invasive/noninvasive techniques. Therefore the objective of this systematic review is to review all recently published animal studies involving noninvasive as well as minimally invasive procedures for acceleration of orthodontic tooth movement.

## 2. Materials and Methods

### 2.1. Eligibility Criteria

Publications included in this study comprised research articles from the past six years, that is, from January 2009 till 31 December 2014. Eligibility criteria for inclusion were original in vivo researches on the noninvasive/minimally invasive modalities to enhance orthodontic tooth movement in animals. Randomized clinical trials and human based researches were excluded from the systematic review. Articles dealing with role of biochemical and cytokines were excluded from the study. Highly invasive procedures like Wilckodontics and periodontally assisted orthodontics were also excluded from this systematic review (Table 2).

### 2.2. Information Resources and Search Strategy

Electronic database was searched in this study with related keyword combinations, using three main search engines to track down the articles.

Electronic databases searched are as follows:

- NCBI databases:
   PubMed.
   PubMed Central.
   PubMed Health.
- MedPilot:
   Medline.
   Catalogue ZB MED.
   Catalogue Medicine Health.
   Excerpta Medica Database (EMBASE).
- Google Scholar.

The main keyword used to search the literature was “orthodontic tooth movement”, which was searched in combination with the following terms:

- *Concerning enhancement of movement*: accelerate, rapid, velocity.
- *Concerning invasiveness*: minimally invasive, non invasive.

### 2.3. Data Extraction and Quality Assessment

Two authors independently searched the literature, selected the studies, extracted the data, and assessed the risk of bias of the studies using ARRIVE (Animal Research: Reporting of In Vivo Experiments) guidelines [26]. Interobserver disagreements were resolved with discussions. The quality assessment of the included studies was performed by using ARRIVE guidelines [26]. Maximum score of 20 was attributed to each study. Studies were evaluated and categorized as good (≥75%), moderate (56% to 74%), or poor (≤55%) quality based on the total score attained (Table 3).

### 2.4. Statistical Analysis

Cohen's kappa analysis was performed to assess the interobserver agreement to grade the quality of the studies, using SPSS version 20. The level of agreement was evaluated by Landis and Koch criteria [27]. Interrater agreement is near to perfect if the value of kappa is 0.81–1, substantial if kappa is 0.61–0.80, moderate if kappa is 0.41–0.60, fair if kappa is 0.21–0.40, and poor if kappa is less than 0.20.

## 3. Results

### 3.1. Study Selection

**Figure 1:**
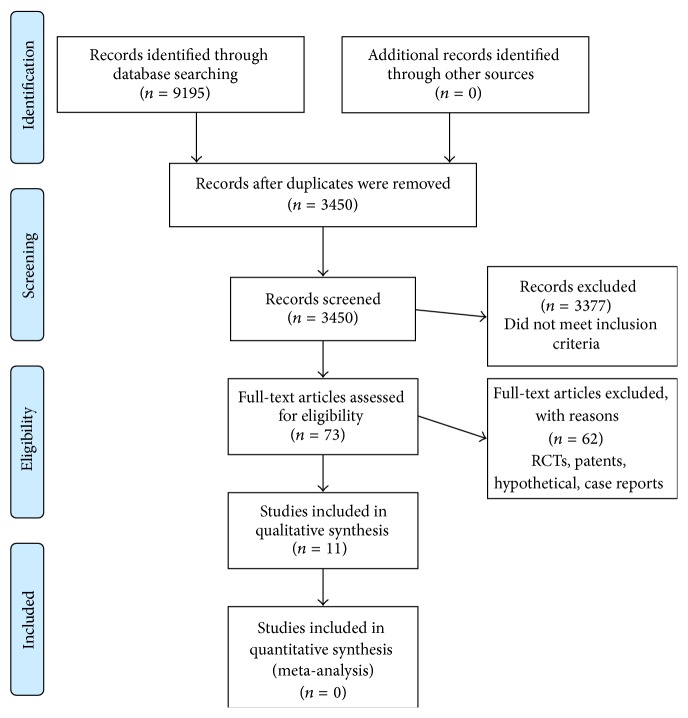
PRISMA 2009 flow diagram. From [60]. For more information, visit http://www.prisma-statement.org/.

PRISMA guidelines were followed to scrutinize the articles as detailed in Table 4 and Figure 1. The total number of hits was 9195 in the databases: 8873 in Google Scholar, 43 in MedPilot, and 279 in NCBI search resources. After adjusting the duplicates, 3450 hits were scrutinized for inclusion in the study. The majority of them were excluded as they did not match the inclusion criteria, leaving 73 publications. After excluding randomized clinical trials, patents, case reports, and hypothetical articles, just 11 original articles were remained which were included in this systematic review.

Interobserver reliability for 20 criteria was 0.54 which is a moderate level of agreement. Cohen's kappa for the majority of the criteria from A to T showed absolute agreement except four criteria which showed moderate-to-good level of interrater agreement: A = 1, B = 0.45, C = 1, D = 0.76, E = 0.58, F = 0.88, G = 0.88, H = 1, I = 0.76, J = 0.87, K = 1, L = 1, M = 0.83, N = 0.90, O = 0.86, P = 0.94, Q = 1, R = 0.82, S = 0.92, and T = 0.90.

### 3.2. Study Characteristics

The selected articles could be categorized in two major categories: (A) studies focusing on noninvasive modalities and (B) studies involving minimally invasive modalities. Noninvasive procedures included 5 articles and studies based on minimally invasive techniques were 5. One article combined both invasive and noninvasive procedure to enhance orthodontic tooth movement.

In noninvasive modalities 2 researches were based on the use of low level laser therapy (LLLT) for acceleration of orthodontic tooth movement, 1 article evaluated mechanical vibration, and 2 involved low intensity pulsed ultrasound (LIPUS). One article studied the effect of LLLT with piezocision on velocity of tooth movement in animal model.

In minimally invasive group, all researches involved flapless corticision with slightly different approaches. Three researchers used piezoelectric knife, 1 author used laser assisted corticision, and 1 research evaluated flapless corticotomy using burs.

### 3.3. Quality Assessment and Risk of Bias

The quality of the studies was assessed by ARRIVE guidelines.  Table 5 shows the assessment of the included studies. The quality of most of the studies was good and none of the studies were categorized as poor quality (Table 3). Low level laser therapy was the most common among noninvasive modalities (2 articles) as it was also used along with corticision in an article. However flapless piezocision was among the commonest minimally invasive procedures to enhance orthodontic tooth movement (3 studies).

## 4. Discussion

### 4.1. Noninvasive Techniques 

#### 4.1.1. Low Level Laser Therapy

Low level laser therapy (LLLT) is also known as photobiomodulation or biostimulation that involves the use of near infrared or low levels of red light to treat a variety of ailments. It does not raise local tissue temperature by more than 1°C and therefore is referred to as “cold laser” or “low level laser” [8, 28]. Although the exact mechanism of therapeutic effects of LLLT is not well established yet, it has been observed that it has effects at the molecular, cellular, and tissue levels. At the cellular level, there is strong evidence that LLLT acts on mitochondria [29] which results in increase in adenosine triphosphate (ATP) production [30] and the induction of transcription factors [31]. These transcription factors trigger protein synthesis leading to cell proliferation and migration. It also modulates the levels of cytokines, inflammatory mediators, and growth factors [32]. Since LLLT accelerate bone regeneration and remodeling by increasing vascularization, promoting trabecular osteoid tissue formation, and enhancing tissue metabolism [33], therefore it was thought to be beneficial also in acceleration of orthodontic tooth movement.

In vitro studies involving rat osteoclast precursor cells and osteoclasts have shown that laser irradiation induces differentiation and activation of osteoclasts [34–38] through expression of RANK, MMP-9, cathepsin K, and *α* (v) *β*3 integrin.

In all of the articles included in this systematic review, diode laser was the source of LLLT including the one which combined LLLT with corticision; however the wavelength, frequency, energy input, and hence the results were slightly different (Table 6) [39, 40]. Shirazi et al. [39] in their research concluded that LLLT can increase the velocity of tooth movement 2.3-fold and the laser light does not reflect to the contralateral side as they found no difference in the movement on the contralateral side compared with the control group. However Altan et al. [40] reported no difference between laser and control groups after application of high energy density. The reason for insignificant results could be the use of higher energy density (54 J) used by Altan in his study, because the most effective range of LLLT for biomodulation is reported to be 0.5–4 J/cm^2^ [41].

Kim et al. applied high energy density laser therapy and found it equally effective in accelerating tooth movement as corticision [42]. But the difference in their research from other reviewed articles was the pulsed mode of laser therapy rather than the continuous mode. When both the procedures (LLLT and corticision) were performed on the same site, there was decrease in the velocity of tooth movement. Although the article was good in quality assessment, the sample size (*n* = 6 premolars in each group) was too small to reach any conclusion.

#### 4.1.2. Low Intensity Pulsed Ultrasound

Ultrasound is a sound wave having frequency above the limit of human ear perception, which can be transmitted into biological tissues. It is widely used in the field of medicine for diagnostic as well as therapeutic purpose [43]. LIPUS stimulation is being utilized effectively as therapeutic modality for bone regeneration and fracture healing; therefore it has been approved by the U.S. Food and Drug Administration (FDA) for healing of fractured bone [8].

Although very limited studies have been conducted on the effects of LIPUS on tooth movement, in vitro studies have shown that LIPUS has anabolic effects on growth factors and other signaling factors production that results in differentiation of osteogenic cells and extracellular matrix [44]. In a very recent study involving rat model, LIPUS accelerated orthodontic tooth movement by 45% and promoted alveolar bone remodeling by stimulating the HGF/Runx2/BMP-2 signaling pathway and RANKL expression [45].

In this systematic review, 2 animal studies related to the role of LIPUS on orthodontic tooth movement were reviewed (Table 7). The outcome of both the researches was different in spite of using the same specification of LIPUS. Xue et al. reported 55%, 37%, and 45% acceleration in tooth movement after application of LIPUS for 5, 7, and 14 days, respectively; however Al-Daghreer et al. found no difference in tooth movement even after application for 4 weeks [45, 46].

### 4.2. Mechanical Vibration

Low level mechanical vibration has profound effect on musculoskeletal morphology [47]. Mechanical vibration signals can promote bone healing, enhance bone strength, and reduce the negative effect of catabolic process [48]. It was hypothesized that mechanical vibration may reduce the lag phase (hyalinization) of orthodontic tooth movement and can result in painless and rapid movement [49].

In this review just 1 article in relation to the use of mechanical vibration for orthodontic tooth movement was reviewed [50]. Shirazi et al. [39] used animal model to assess the effect of mechanical vibration on incisor's movement and reported favorable results but because of vague methodology, indistinct selection criteria, and moderate quality scores, it was difficult for us to give remarks on the effect of mechanical vibration on orthodontic tooth movement.

### 4.3. Minimally Invasive Techniques

Osteotomy and corticotomy to accelerate tooth movement is not new in orthodontics, introduced by Köle in 1959 [51]. His concept was to segment the teeth containing alveolar bone with lingual and labial osteotomy and move the whole segmented alveolus with orthodontic forces. The technique was effective but required buccal as well as lingual full-thickness flaps followed by massive decortication of alveolar bone on buccal and lingual sides making the procedure very invasive and painful [52]. Thus the acceptance of the procedure was low and researchers were always looking for less invasive methods. Since the rapid movement in the procedure is not due to en bloc movement of alveolus rather there is a mechanism of accelerated soft tissue and hard tissue remodeling “Regional Acceleratory Phenomenon (RAP)” associated directly with the severity of surgical procedure [53]. Osteoperforations placed even far from the tooth to be moved can increase the rate of tooth movement, by increasing the level of inflammatory cytokine expression and extensive osteoporotic changes [54]. This led to the incessant development of less invasive approaches.

In this systematic review, five animal studies in relation to the minimally invasive technique to accelerate orthodontic tooth movement were reviewed. Mucoperiosteal flap was not reflected in any of the researches and there was no massive decortication of cortical bone, which made the procedures less invasive. In two studies, piezosurgery unit was used to perform cuts on the buccal alveolar bone, mesial and distal to the tooth to be moved [55, 56]. However Xue et al. used ultrasonic piezotome to create multiple holes buccally and lingually [45]. Since the velocity of tooth movement is in direct proportion to the amount of surgical insult, Ruso et al. [56] found acceleration only by 135% which was though significantly greater than the conventional group but lesser than the corticotomy induced acceleration reported earlier [57]. This was in accordance with the ultrasonic piezopuncture method used by Xue et al. [45] who suggested repeated application at regular intervals to overcome the deficient RAP phase associated. On the other hand Teixeira et al. [54] concluded that greater increase in velocity of tooth movement can be obtained if mechanical stimulation of alveolar bone is maintained through constant orthodontic force, along with piezosurgery. Seifi et al. [58] used Er,Cr;YSGG laser device with the energy range of 300 mJ and pulse rates of 20 Hz for corticotomy. They found twofold acceleration in tooth movement, without any adverse effects on periodontal health on the experimental side. Safavi et al. [59] used tungsten carbide bur in high torque slow speed surgical handpiece to make holes in the buccal cortical plate. They found accelerated tooth movement in the first month of the experiment followed by lesser amount of movement in the third month of experiment. The reason could be the formation of more mature lamellar bone after bur decortication as compared to the control group (Table 8).

## 5. Conclusion

It can be concluded from the study that LLLT and flapless corticotomy have some evidence of accelerating effect on orthodontic tooth movement; however there is no set protocol found for the procedures yet. LIPUS and mechanical vibrations are also emerging noninvasive modalities but due to fewer studies, no evidence based conclusion can be drawn.

## Figures and Tables

**Table 1:** Methods to reduce orthodontic treatment duration.

More efficient mechanics	(i) Low friction mechanics (ii) Self-ligating brackets (iii) Preformed robotic archwires (iv) Microimplants

Enhance bone remodeling	(i) Biochemical (ii) Parathyroid hormone (iii) Parathyroid hormone (iv) Osteocalcin (v) Dihydroxyvitamin D3 (1,25-(OH)2D3)

Physical stimulation	(i) Micropulse and cyclic vibration (ii) Low level laser therapy (iii) Low intensity pulsed ultrasound

Surgical approach	(i) Corticotomy (ii) Periodontally assisted osteogenic orthodontics (iii) Piezocision assisted orthodontics

**Table 2:** Inclusion and exclusion criteria for the systematic review.

Inclusion criteria	Exclusion criteria
Original research articles referring to noninvasive modalities or minimally invasive techniques to accelerate orthodontic tooth movement Animal studies	Randomized clinical trials Articles dealing with highly invasive procedures Articles referring to use of biochemical or drugs to accelerate tooth movement Microimplants or frictionless brackets as a modality to reduce treatment duration Reviews, interviews, and discussions

**Table 3:** Quality assessment scores of selected studies.

Procedure	Good	Moderate	Poor
	≥75%	56% to 74%	≤55%
Minimally invasive [5]	4	1	
Noninvasive [5]	4	1	
Combination [1]	1		
	9	2	

**Table 4:** PRISMA 2009 Checklist.

Section/topic	#	Checklist item	Reported on page #
Title			
Title	1	Identify the report as a systematic review, meta-analysis, or both	1
Abstract			
Structured summary	2	Provide a structured summary including, as applicable, background; objectives; data sources; study eligibility criteria, participants, and interventions; study appraisal and synthesis methods; results; limitations; conclusions and implications of key findings; systematic review registration number	1
Introduction			
Rationale	3	Describe the rationale for the review in the context of what is already known	3
Objectives	4	Provide an explicit statement of questions being addressed with reference to participants, interventions, comparisons, outcomes, and study design (PICOS)	3
Methods			
Protocol and registration	5	Indicate if a review protocol exists and if and where it can be accessed (e.g., Web address) and if available provide registration information including registration number	
Eligibility criteria	6	Specify study characteristics (e.g., PICOS, length of follow-up) and report characteristics (e.g., years considered, language, and publication status) used as criteria for eligibility, giving rationale	3
Information sources	7	Describe all information sources (e.g., databases with dates of coverage, contact with study authors to identify additional studies) in the search and date last searched	3
Search	8	Present full electronic search strategy for at least one database, including any limits used, such that it could be repeated	4
Study selection	9	State the process for selecting studies (i.e., screening, eligibility, included in systematic review and if applicable included in the meta-analysis)	4
Data collection process	10	Describe method of data extraction from reports (e.g., piloted forms, independently, in duplicate) and any processes for obtaining and confirming data from investigators	4
Data items	11	List and define all variables for which data were sought (e.g., PICOS, funding sources) and any assumptions and simplifications made	
Risk of bias in individual studies	12	Describe methods used for assessing risk of bias of individual studies (including specification of whether this was done at the study or outcome level) and how this information is to be used in any data synthesis	4
Summary measures	13	State the principal summary measures (e.g., risk ratio, difference in means)	
Synthesis of results	14	Describe the methods of handling data and combining results of studies, if done, including measures of consistency (e.g., *I* ^2^) for each meta-analysis	
Risk of bias across studies	15	Specify any assessment of risk of bias that may affect the cumulative evidence (e.g., publication bias, selective reporting within studies)	4
Additional analyses	16	Describe methods of additional analyses (e.g., sensitivity or subgroup analyses, metaregression), if done, indicating which were prespecified	4
Results			
Study selection	17	Give numbers of studies screened, assessed for eligibility, and included in the review, with reasons for exclusions at each stage, ideally with a flow diagram	5
Study characteristics	18	For each study, present characteristics for which data were extracted (e.g., study size, PICOS, and follow-up period) and provide the citations	
Risk of bias within studies	19	Present data on risk of bias of each study and if available any outcome level assessment (see item 12)	6
Results of individual studies	20	For all outcomes considered (benefits or harms), present, for each study: (a) simple summary data for each intervention group, (b) effect estimates and confidence intervals, ideally with a forest plot	
Synthesis of results	21	Present results of each meta-analysis done, including confidence intervals and measures of consistency	
Risk of bias across studies	22	Present results of any assessment of risk of bias across studies (see item 15)	6
Additional analysis	23	Give results of additional analyses, if done (e.g., sensitivity or subgroup analyses, metaregression [see item 16])	
Discussion			
Summary of evidence	24	Summarize the main findings including the strength of evidence for each main outcome; consider their relevance to key groups (e.g., healthcare providers, users, and policy makers)	6–10
Limitations	25	Discuss limitations at study and outcome level (e.g., risk of bias) and at review-level (e.g., incomplete retrieval of identified research, reporting bias)	
Conclusions	26	Provide a general interpretation of the results in the context of other evidence and implications for future research	10
Funding			
Funding	27	Describe sources of funding for the systematic review and other support (e.g., supply of data); role of funders for the systematic review	Nil

From [60]. For more information, visit: http://www.prisma-statement.org/.

**Table 5:** Assessment of the included studies based on quality assessment tool.

Author	Year	Topic	1	2	3	4	5	6	7	8	9	10	11	12	13	14	15	16	17	18	19	20	Score
Altan et al. [40]	2012	LLLT	✓	✓	✓	✓	✓	✓	✓	✓	✓	✓ *✗*	✓	✓	✓ *✗*	✓	✓	*✗*	*✗*	✓ *✗*	✓ *✗*	✓	16
Shirazi et al. [39]	2013	LLLT	✓	✓	✓	✓	✓	✓	✓	✓	✓	✓ *✗*	✓ *✗*	✓	✓	✓	✓	✓	*✗*	✓	✓	*✗*	17
Xue et al. [45]	2013	LIPUS	✓	✓	✓	✓	✓	✓	✓		✓	✓ *✗*	✓ *✗*	✓	✓	✓	✓	✓	✓ *✗*	✓ *✗*	*✗*	✓	17
Al-Daghreer et al. [46]	2014	LIPUS	✓	✓	✓	✓	✓	✓	✓	*✗*	*✗*	✓ *✗*	✓	✓	✓	*✗*	✓	✓ *✗*	*✗*	✓	✓	✓	15
AlSayagh and Salman [50]	2014	Mechanical vibration	✓	*✗*	✓	*✗*	*✗*	✓	✓	*✗*	✓	✓ *✗*	*✗*	*✗*	✓	✓	✓	*✗*	*✗*	✓	✓	*✗*	10
Kim et al. [42]	2009	LLLT and corticision	✓	✓	✓	✓	*✗*	✓	✓	✓	✓	✓ *✗*	✓	✓	✓	✓	✓	✓	*✗*	✓	✓	✓	18
Seifi et al. [58]	2012	Laser assisted flapless corticotomy	✓	✓	✓	✓	*✗*	✓ *✗*	✓	*✗*	*✗*	✓ *✗*	*✗*	✓	*✗*	*✗*	✓	*✗*	✓	✓	✓	*✗*	11
Kim et al. [61]	2013	Piezopuncture	✓	✓	✓	✓	*✗*	✓	✓ *✗*	✓	✓	✓ *✗*	✓	✓	✓	✓	✓	✓ *✗*	*✗*	✓ *✗*	✓	✓	16
Safavi et al. [59]	2012	Flapless bur decortication	✓	✓ *✗*	✓	✓	*✗*	✓	✓	✓	*✗*	✓ *✗*	*✗*	*✗*	✓	✓	✓	✓	✓	✓	✓	✓	15
Dibart et al. [55]	2013	Piezocision	✓	✓	✓	*✗*	✓	✓	✓	✓	*✗*	✓ *✗*	*✗*	✓	*✗*	✓	✓	✓ *✗*	✓	✓	✓	✓	15
Ruso et al. [56]	2013	Flapless decortication	✓	✓ *✗*	✓	✓	✓	✓	✓	✓	*✗*	✓ *✗*	✓	✓	✓	✓	✓	✓	✓	✓	✓	✓	18

**Table 6:** Use of LLLT to accelerate orthodontic tooth movement in animals.

Author name	Sample	Laser type	Energy	Results	Movement in experimental group (mm)	Movement in control group (mm)
Shirazi et al. [39]	30 rats divided into 2 groups, 15 each	GaAlP diode 660 nm Continuous wave mode 25 mW 660 nm	7.5 J/session 5 min/session after every 48 hrs for a total of 6 sessions	2.3-fold acceleration in tooth movement in laser irradiated group	0.39 ± 0.07 *P* < 0.001	0.11 ± 0.04

Altan et al. [40]	38 male Wistar rats divided into 4 groups: 3 experimental groups = 11 rats each, 1 control group = 5 rats	GaAlAs 820 nm Continuous mode 100 mW	One group received 54 J/session The other group received 15 J/session applied daily for 8 days	No statistically significant result	Not mentioned	Not mentioned

Kim et al. [42] (combination with corticision)	12 beagle dogs Maxillary 2nd premolars (*n* = 24) divided into 4 groups (*n* = 6) Split mouth design	GaAlAs 808 nm Pulsed mode 763 mW	75 mJ per pulse 41.7 J/cm^2^/point 333.6 J/cm^2^/session Applied every 3rd day for 8 weeks	LLLT accelerated tooth movement 3.75-fold Corticision accelerated tooth movement 3.76-fold No significant difference in tooth movement in LLLT + corticision group	4.62 ± 0.25 *P* < 0.001 4.61 ± 0.30 *P* < 0.001 0.88 ± 0.19 *P* < 0.001	0.23 ± 0.18

**Table 7:** Use of low intensity pulsed ultrasound and mechanical vibrations to accelerate tooth movement in animals.

Author	Sample	LIPUS and vibration specification	Duration	Results	Movement in experimental group	Movement in control group
Xue et al. [45]	48 rats divided into 6 groups	Frequency 1.5-MHz; intensity 30 mW/cm^2^	Burst of 200 *µ*s followed by pause of 800 *µ*s 20 min/day for 14 days	55%, 36%, and 45% acceleration in tooth movement on days 5, 7, and 14, respectively	1118 *µ*m ± not given	773 ± not given

Al-Daghreer et al. [46]	10 beagle dogs Split mouth design	Frequency 1.5 MHz; intensity 30 mW/cm^2^	200 *μ*s 20 min/day for 4 weeks	No significant difference in the amount of tooth movement	0.79 mm ± 0.17 *P* = 0.05	0.6 mm ± 0.21

AlSayagh and Salman [50]	14 rabbits divided into 2 groups (*n* = 7)	Frequency 113 Hz	1, 3, 5, 8, 10, 12, 15, 17, and 19 days (9 sessions of 10 min each in 22 days)	Acceleration in orthodontic tooth movement	3.73 mm ± 0.24	3.11 mm ± 0.07

**Table 8:** Use of flapless corticotomy to accelerate orthodontic tooth movement in animals.

Author	Sample	Procedure	Duration of study	Results	Movement in experimental group (mm)	Movement in control group (mm)
Dibart et al. [55]	94 Sprague Dawley rats divided into 4 groups: control = 3, tooth movement = 21, piezocision = 35, and piezocision + tooth movement = 35	Flapless piezocision	56 days	Tooth movement accelerated 2-fold	Not mentioned	Not mentioned

Ruso et al. [56]	6 dogs Split mouth design	Flapless piezocision and expansion with archwire	9 weeks followed by 2 weeks of consolidation	135% acceleration in tooth movement	21.9 ± 8.1° *P* < 0.05	10.7±6°

Kim et al. [42]	10 dogs Control (*n* = 4), experimental (*n* = 6)	Flapless piezopuncture	6 weeks	Tooth movement accelerated 3.26- and 2.45-fold in maxilla and mandible, respectively	2.31 ± 0.82 *P* < 0.05 1.33 ± 0.28 *P* < 0.05	0.72 ± 0.06 in maxilla 0.51 ± 0.19 in mandible

Safavi et al. [59]	5 dogs Split mouth design	Flapless bur decortication	3 months	No significant difference in tooth movement	4.59 ± 2.45 *P* = 0.063	4.88 ± 1.93

Seifi et al. [58]	8 rabbits Split mouth design	Flapless (Er-Cr:YSGG) laserassisted corticotomy	21 days	1.77-fold acceleration in tooth movement	1.65 ± 0.34 *P* = 0.001	0.93 ± 0.28

## Abbreviations

LLLT: Low level laser therapy
LIPUS: Low intensity pulsed ultrasound.
